# Critical analysis of molluscicide application in schistosomiasis control programs in Brazil

**DOI:** 10.1186/s40249-016-0153-6

**Published:** 2016-07-04

**Authors:** PMZ Coelho, RL Caldeira

**Affiliations:** Research group of Schistosoma mansoni Biology and Its Interaction with the Host, René Rachou Institute, Oswaldo Cruz Foundation-Minas Gerais, 30190-002 Belo Horizonte, MG Brazil; Research group of Medical Helminthology and Malacology, René Rachou Institute, Oswaldo Cruz Foundation-Minas Gerais, 30190-002 Belo Horizonte, MG Brazil

**Keywords:** *Biomphalaria*, Molluscicide, *Schistosoma mansoni*, Schistosomiasis, Vector control, Brazil

## Abstract

In Brazil, *Biomphalaria glabrata*, *B. tenagophila*, and *B. straminea* are naturally infected by the trematode *Schistosoma mansoni*, the causative agent of schistosomiasis. Despite decades of governmental efforts through official control programs, schistosomiasis remains an important public health problem in the country: thousands of people are infected with the trematode each year and millions live in endemic areas. The World Health Organization recommends using a combination of molluscicide (niclosamide) and mass chemotherapy to control the transmission of schistosomiasis, with this treatment successfully reducing the morbidity of the disease. In the past, niclosamide has been used in official schistosomiasis control programs in Brazil. However, as *B. glabrata* recolonizes even after molluscicide application, the use of molluscicides has gradually decreased in the country until they were discontinued in 2002, mainly due to the rising global pressure to preserve the environment and the difficulties of obtaining licenses from the Brazilian Ministry of Environment to use toxic substances in aquatic ecosystems. Therefore, the discovery of new molluscicides, which could be more selective to *Biomphalaria* species and less harmful to the aquatic ecosystem, is necessary. In addition, political efforts to sensitize funders to provide grants for this field of research are required. In this context, this article aims to make a critical analysis of molluscicide application in schistosomiasis control programs in Brazil.

**Electronic supplementary material:**

The online version of this article (doi:10.1186/s40249-016-0153-6) contains supplementary material, which is available to authorized users.

## Multilingual abstracts

Please see Additional file 1 for translations of the abstract into the six official working languages of the United Nations

## Background

Despite decades of governmental efforts through official control programs, schistosomiasis remains an important public health problem in the country: thousands of people are infected with the trematode each year and millions live in endemic areas. The World Health Organization recommends using a combination of molluscicide (niclosamide) and mass chemotherapy to control the transmission of schistosomiasis, with this treatment successfully reducing the morbidity of the disease. In Brazil, as *Biomphlaria glabrata* recolonizes even after molluscicide application, the use of molluscicides has gradually decreased in the country. However, the discovery of new molluscicides is necessary, especially, which could be more selective to *Biomphalaria* species and less harmful to the aquatic ecosystem.

The trematode *Schistosoma mansoni* Sambon, 1907 is the causative agent of schistosomiasis, which is an important public health problem in Brazil. Even after decades of governmental efforts through official control programs, thousands of people are still infected with the trematode and millions live in endemic areas. *S. mansoni* is transmitted by *Biomphalaria* snails, which act as intermediate hosts. Snail control by means of molluscicides is considered an auxiliary method within integrated control of schistosomiasis. Between 1946 and 1955, ~7000 chemical products were tested as potential molluscicides [1]. Among these, niclosamide (2-amino ethanol salt of 2’, 5’-dichloro-4’-nitro salicylanilide; Bayluscide®, Bayer, Germany) has been recommended by the World Health Organization (WHO) as a molluscicide since the 1960s [2], and it is still the molluscicide of choice for controlling the transmission of schistosomiasis today [3].

However, it is worth noting this mollusc has been present since the Jurassic period (160 Ma ago), according to geological records in Europe and the United States [4], which demonstrates its adaptive ability. These planorbids generally do not survive for more than one year and are prolific in nature. Their persistence stems from their reproduction rate, which depends on other intrinsic ecological factors that influence their fertility, egg laying, and viability. Although these snails are hermaphrodites and can self-fertilize, they prefer to reproduce by cross-fertilization when paired. This proliferation and self-fertilization make population control in these snails difficult because one snail can produce 10 million descendants in just 3 months [5, 6]. An important peculiarity of these organisms is their capacity to resist slow desiccation and survive for more than 6 months in spite of a high mortality rate [7]. In addition to host resistance, the parasite in the primary sporocyst phase can undergo temporary suspension of development, consequently decreasing its metabolism and saving vital energy [8, 9]. Upon return of favorable conditions, the survivors rebuild the colonies (founder effect) and become the link between the previous and subsequent populations [5, 10], while the parasites return to their normal development and shed their cercariae [8, 9].

**Fig. 1:**
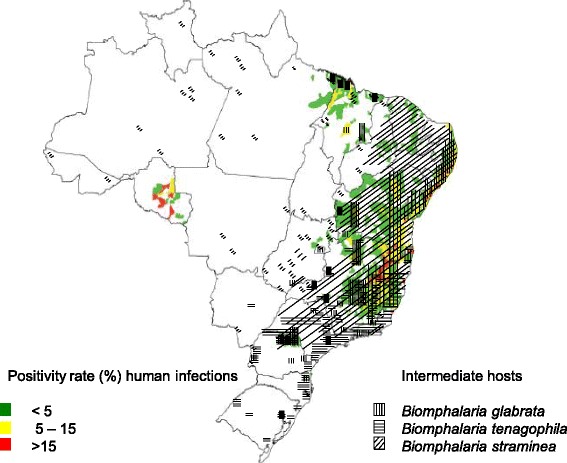
Distribution of positive *Schistosoma mansoni* cases (source: Ministério da Saúde, 2014) and their intermediate hosts (adapted from Paraense [12]) in Brazil

This knowledge is important for understanding the conditions and circumstances of schistosomiasis and its epidemiological characteristics in each region. Transmission levels depend on *Biomphalaria* species present in the focus, and the degree of compatibility between the snail species and the strain of *S. mansoni* that infects the local human population [11]. In Brazil, there are 11 *Biomphalaria* species, however, only *Biomphalaria glabrata*, *B. tenagophila*, and *B. straminea* maintain the *S. mansoni* cycle in several states [12], with different positivity rates [13] (see Fig. 1).

### Use of molluscicides in China and globally

In two previous meetings, the WHO Expert Committee on Bilharziasis recommended the use of molluscicides as an important measure to control schistosomiasis. The first WHO report from 1953 [14] recommended the use of molluscicides with other traditionally used control measures such as chemotherapy, provision of basic sanitation, water treatment, and health education. The second report, published in 1961, concluded that the molluscicide application to combat intermediate hosts was the most efficient isolated measure to control schistosomiasis [2].

In China, before the 1950s, some synthetic molluscicides, such as sodium pentachlorophenate (NaPCP), were widely used but safety concerns and severe environmental pollution led to NaPCP being banned [15]. Following this, the Chinese government initiated widespread disease control programs using mass chemotherapy in highly endemic regions and selective chemotherapy in areas of medium and low endemicity linked with snail control through mollusciciding and environmental modification, such as cementing canals, re-adjusting irrigation systems, etc. [16]. This resulted in the reduction of the prevalence and morbidity of the disease reaching their lowest levels between 1989 and 1995 of close to 50 % in human, and 32 % in livestock such as cattle and buffalo [16, 17]. Unfortunately, just after the end of the control program in China in 2001, the disease emerged or reemerged in some regions [18], and a new integrated control program was launched in 2004, with an emphasis on health education, access to clean water and adequate sanitation, agricultural mechanization, fencing of water buffaloes, and chemotherapy [19, 20].

Currently, the molluscicide used in China is niclosamide, which has been recommended by the WHO since the 1960s [2]. Yang et al. [21] conducted a systematic review and meta-analysis to assess the molluscicidal effects of the currently recommended combination of 50 % niclosamide ethanolamine salt wettable powder and a new 4 % niclosamide ethanolamine salt powder developed by He et al. [22]. They observed that both are good enough to be used as molluscicides integrated into a schistosomiasis control program and that application more than twice annually was necessary.

King and Bertsch [23] showed in a historical retrospective focusing specifically on niclosamide that snail control is an effective means for reducing local *Schistosoma* transmission in several countries, such as Saint Lucia, Iran, Tunisia, Morocco, Saint Kitts and Egypt. When properly implemented, snail control is an auxiliary tool in modern mass drug delivery programs, resulting in improved prevention of *Schistosoma* infection and reinfection. King et al. [24] carried out a systematic review and meta-analysis to summarize prior experiences of molluscicide-based control of *Bulinus* and *Biomphalaria* spp. snails, and estimated its impact on local human *Schistosoma* infection. They concluded that regular focal molluscicide application contributed significantly towards the elimination of schistosomiasis in high-risk areas.

### Use of molluscicides in Brazil

In Brazil, until the 1970s, various biological and chemical compounds were tested and used as molluscicides. One of these was *Bacillus pinotti*, Cruz and Dias 1953, isolated from the ovotestis of *B. glabrata*. These bacteria are proteolytic aerobic organisms that are harmless to several vertebrate species and, under certain conditions, lethal to *B. glabrata* and other molluscs [25–27]. Studies focusing on their use in the biological control of some intermediate hosts [27] have been conducted, however, the results of these were not considered definitive [28]. Various chemical compounds, although considered toxic to the environment, have been used in experimental field studies [29], including copper sulfate, sodium pentachlorophenate, tritylmorpholine, and niclosamide. After 1970, niclosamide has been the molluscicide of choice in official schistosomiasis control programs in Brazil.

Barbosa and Costa [30], who used monthly molluscicide application as a unique measure to control schistosomiasis in a long-term study, reported a decrease in the prevalence of the disease. Pieri et al. [31] obtained similar results in an endemic area in Northeast Brazil. The combination of molluscicide application and chemotherapy showed the best results for reducing the prevalence of the disease in a short period of time. However, as *B. glabrata* recolonizes even after molluscicide application, this is one of the disadvantages of molluscicide use.

After 1986, the use of niclosamide progressively decreased until 2001, when only 10 instances of molluscicide applications using niclosamide were reported (see Table 1). Although the data are related only to some Northeastern Brazilian states (Ceará, Rio Grande do Norte, Paraíba, Pernambuco, Alagoas, and Sergipe), they are considered to be representative of molluscicide use throughout Brazil [32] and show the important role of molluscicides in the control of *S. mansoni*. Unfortunately, to the best of our knowledge, no studies have been conducted showing the impact of using molluscicides in Brazil, which hinders the measurement of their success. Nevertheless, isolated data, from 1978 to 1986, showed that the prevalence of the disease decreased [29, 31]. After 2002, the Brazilian Ministry of Health did not receive any more reports of niclosamide use, mainly because of the increasing global pressure to preserve the environment. Subsequently, obtaining licenses from the Brazilian Ministry of the Environment to use molluscicides was difficult. After pressure from the Ministry of Agriculture and the Ministry of Health, new instructions to control agriculture plagues and human disease vectors were issued. Thus, in 2006, the Ministry of Agriculture and Public Health agencies obtained a license from the Ministry of the Environment that allowed them to decide upon the use of toxic products in special conditions (Instrução Normativa n° 109–3 de Agosto de 2006, Artigo 4 paragrafo 1°, Ministério do Meio Ambiente). Currently, the use of niclosamide is restricted to active foci and in well-defined areas with high prevalence rates of schistosomiasis.

**Table 1:** Number of freshwater habitats surveyed and the percentage of those that had niclosamide (Bayluscide®) application in official schistosomiasis control programs in Northeastern Brazilian states (Ceará, Rio Grande do Norte, Paraíba, Pernambuco, Alagoas, and Sergipe) in 1976–2002

Year	Number of freshwater habitats surveyed	Niclosamide application (%)
1976	16 488	11.4
1977	43 178	3.4
1978	106 319	19.2
1979	54 817	32.4
1980	126 272	26.6
1981	123 070	23.5
1982	139 255	19.6
1983	178 178	21.0
1984	197 337	14.0
1985	212 113	14.4
1986	156 183	11.0
1987	55 339	11.1
1988	79 287	6.8
1989	100 720	7.1
1990	37 400	11.4
1991	51 012	8.5
1992	50 507	14.8
1993	45 572	15.7
1994	31 772	14.8
1995	34 181	13.7
1996	31 183	8.3
1997	27 832	14.4
1998	44 28	34.8
1999	7 774	9.4
2000	1 519	19.8
2001	3 306	0.3
2002	2 871	0

Source: Secretary of Health Surveillance, Brazilian Health Ministry

It has also been shown that niclosamide can reduce the population of snails and make a substantial impact on the prevalence and incidence of human schistosomiasis infection (when used together with treatment strategies). A 10 % decrease in the prevalence of schistosomiasis was reported in the 1950s [33]. However, molluscicides have some disadvantages, including not being cost-effective [34], having lethal effects on other organisms [35], and requiring frequent application [30, 31, 36]. A recent schistosomiasis prevalence survey of all Brazilian states, in which 220,000 fecal samples from students aged 7–14 years were tested by analyzing two slides using the Kato-Katz technique [37], revealed an estimated prevalence of more than one million people infected with schistosomiasis throughout the country [38]. However, this prevalence was likely underestimated because most Brazilian endemic areas have low prevalence rates of schistosomiasis and most of the infected population has a low worm load that is difficult to detect using the Kato-Katz technique. To test this, a study involving meticulous screening of the infected population was performed by analyzing 18 slides (as compared to one, the method adopted by official Brazilian control program) using the Kato-Katz technique in areas with low prevalence and low infection intensity. The results showed that the prevalence obtained by examining one Kato-Katz slide was 8 % compared with the prevalence of 35.8 % obtained from examining 18 slides [39, 40].

Considering the current Brazilian situation, we believe that evaluating the use of molluscicides in order to decrease schistosomiasis transmission in some endemic areas is now necessary. However, it is important to consider each endemic focus with its specific characteristics, such as the size of the transmission areas to be treated, environmental impacts and especially, the diverse, natural and artificial habitats (lakes, dams, river streams, and irrigation ditches) of *Biomphalaria*.

### Alternatives for snail control

A promising model to control schistosomiasis transmission is introducing *B. tenagophila* Taim lineage, which is totally resistant to infection by *S. mansoni*, in areas where the transmission of schistosomiasis is only due to this *Biomphalaria* species. *B. tenagophila* is able to transfer that resistance as a dominant characteristic to its descendants by cross-breeding with susceptible snails. The model can be improved by using molluscicides before introducing *B. tenagophila-*resistant (Taim strain) snails [41].

Efforts are being made to discover molluscicidal products of plant origin, especially potentially biodegradable in nature, due to the growing awareness of environmental pollution. Many plant species, such as *Phytolacca dodecandra* [42], *Alternanthera sessilis* [43], *Jatropha curcas* [44], *Euphorbia royleana*, *E. antisyphilitica*, *E. lactea* ‘Cristata’, *E. pulcherrima*, *E. neutra*, *Croton tiglium*, *Codiaeum variegatum* [45], and *Solanum xanthocarpum* [46], have been demonstrated to have molluscicidal properties against different snail species. In Brazil, Schall et al. [47] reported the potential of latex from the plant *E. splendens*, imported from Madagascar, of being an efficient molluscicidal product, as it is less toxic to other aquatic organisms when compared with niclosamide. The latex produced by this plant was considered by Mott [48] as one of the most potent molluscicides among thousands of natural products obtained from plants. However, more studies need to be conducted to validate the application of *E. splendens*, as well as a cost analysis of these applications compared to niclosamide.

Another study performed in Brazil showed that a potent molluscicide extracted from a tropical plant species (*Piplartine*), which also has less environmental toxicity than niclosamide, is effective against *B. glabrata* [49]. Recentely, Kiros et al. demonstrated that *Glinus lotoides* has molluscicidal activity against *B. pfeifferi* snails and cercariacidal activity against *S. mansoni* trematodes [45]. However, all these studies are preliminary and none of these products have been recognized or authorized for use to date.

In China, some molluscicides have also been reported as effective and less toxic, such as the suspension concentrate of niclosamide [50]. A promising study was conducted by Xia et al. [51] using a novel molluscicide derivative from niclosamide, named LDS (salt of quinoid-2′, 5-dichloro-4′-nitrosalicylanilide). To assess the effects of large-scale field applications of LDS, tests were done in 15 counties in the endemic *S. japonica* areas of Hubei province, China. However, despite showing good results of mollusc mortality, these studies also need to clarify its impact on the environment [51].

Recently, Duval et al. [52] isolated and characterized a new microbial pathogen from *B. glabrata*, and classified it into the *Paenibacillus* genus. These bacteria invade most snail tissues and proliferate, causing high lethality, and can be transmitted both vertically and horizontally to other snails causing them to die within 30 days. However, extensive studies are necessary to evaluate the potential of these bacteria to infect other aquatic organisms and/or cause severe environmental pollution.

## Conclusions

The discovery of new molluscicides, which are more selective to *Biomphalaria* and *Bulinus*, and less harmful to aquatic ecosystems, besides political efforts to sensitize funder to offer grants for this field of research, are necessary.

It is also important to consider that Brazil is a country measuring more than eight million km^2^ and has great variation in schistosomiasis transmission, which requires decisions concerning human safety procedures to appropriate application of molluscicides. Finally, it is important to emphasize that sanitation and water treatment are the most important measures to control schistosomiasis, in addition to other public health diseases such as gastroenteritis and viral hepatitis. Implementing these measures will yield permanent benefits to the entire human population.

## Abbreviations

NaPCP, sodium pentachlorophenate; WHO, World Health Organization.

## Additional file

Additional file 1: Multilingual abstract in the six official working languages of the United Nations. (PDF 665 kb)
