# Supplementary material for: Critical analysis of molluscicide application in schistosomiasis control programs in Brazil
Source: Infect Dis Poverty. 2016 Jul 4;5:57. doi: 10.1186/s40249-016-0153-6 (PMC4931695; doi:10.1186/s40249-016-0153-6)
Supplement: Additional file 1: — Multilingual abstract in the six official working languages of the United Nations. (PDF 665 kb) [file 40249_2016_153_MOESM1_ESM.pdf]

Translation of the abstract into the six official working languages of the United Nations

تحليل هامّ حول استخدام مبيد الرخويات ضمن برامج مراقبة داء البلهارسيات في البرازيل

باولو ماركوس زيش كويلو ورافائيل كلديرا

ملخّص

يُصاب هذا النوع من الحلزونات *Biomphalaria glabrata* ، *B. tenagophila* و*B. straminea* بشكل طبيعي بالمثقوبات من نوع *Schistosoma mansoni* وهي العنصر المسبب لمرض البلهارسيات (schistosomiasis). بالرغم، من المجهودات التي قامت بها الحكومة منذ عقود للتخلص منه اعتمادا على برامج المراقبة الرسمية إلا أن هذا المرض ما يزال يمثل مشكلة صحية بالبلاد: يصاب الآلاف من الناس بالمثقوبات كل عام ويعيش الملايين في مناطق موطونة. تُوصي منظمة الصحة العالمية باستخدام مزيج من مبيد الرخويات (نيكلوزاميد) والعلاج الكيميائي للسيطرة على انتقال داء البلهارسيات وقد نجح هذا العلاج في التقليل من عدد الوفيات جراء هذا المرض. في الماضي، تم استعمال نيكلوزاميد في برامج المراقبة الرسمية لمكافحة انتشار البلهارسيات في البرازيل. ومع ذلك، حتّى بعد استخدام مبيد الرخويات سيظل *B. glabrata* في انتشار. يشهد معدل استخدام هذا المبيد انخفاضا تدريجيا بالبلاد إلى أن توقف استعماله سنة 2002، ويرجع ذلك بالأساس إلى الضغط العالمي المتزايد للحفاظ على البيئة إضافة إلى صعوبة الحصول على ترخيص من وزارة البيئة البرازيلية لاستخدام المواد السامة في الأنظمة البيئية المائية. لذلك من الضروري اكتشاف مبيد جديد للرخويات أفضل لاختيار صنف *Biomphalaria* وأقل ضررا للنظام البيئي المائي. وبالإضافة إلى ذلك، يجب بذل جهود سياسية لتوعية الممولين لتوفير المنح من أجل الأبحاث. وفي هذا السياق، يهدف هذا المقال إلى تقديم تحليل هام لاستعمال مبيد الرخويات ضمن برامج مراقبة انتشار البلهارسيات في البرازيل.

Translated from English version into Arabic by Zeineb Trabelsi, through

TRANSLATORS
WITHOUT BORDERS

巴西血吸虫病防治项目中灭螺药应用的关键点分析

PMZ Coelho and RL Caldeira

摘要

在巴西，光滑双脐螺（*Biomphalaria glabrata*）、浅滩双脐螺（*B. tenagophila*）和藁杆双脐螺（*B. straminea*）可以自然感染曼氏血吸虫（*Schistosoma mansoni*），后者引起血吸虫病。尽管几十年来政府通过官方的防控项目一直在努力防治血吸虫病，该病仍是巴西重要的公共卫生问题。上百万人生活在流行区，每年有数千人被感染。世界卫生组织推荐联合应用灭螺药（氯硝柳胺）和大规模化疗来控制血吸虫病，可以成功降低血吸虫病的发病率。过去，巴西官方的血吸虫病防治项目曾使用氯硝柳胺。但是，应用氯硝柳胺后光滑双脐螺可以很快繁殖，因此灭螺药在巴西的使用逐渐减少，直到 2002 年完全停用，主要是由于日益增长的全球环境保护压力和无法从巴西环保部获得在水体使用有毒物质的许可。因此，研发新的对双脐螺更具特性的且对水体生态系统危害小的灭螺药非常重要。此外，需要从政策层面努力以刺激投资者向现场研究方面投资。基于此，本文对巴西血吸虫病防治规划中灭螺药应用的关键点进行了分析。

Translated from English version into Chinese by Qian Men-Bao, edited by Yang Pin, through

CHINESE CENTER FOR DISEASE CONTROL AND PREVENTION
NATIONAL INSTITUTE OF PARASITIC DISEASES

Analyse critique de l’utilisation de molluscicides dans les programmes de lutte contre la schistosomiase au Brésil

PMZ Coelho et RL Caldeira

Résumé

Au Brésil, *Biomphalaria glabrata*, *B. tenagophila* et *B. straminea* sont infectés naturellement par le trématode *Schistosoma mansoni*, agent de la schistosomiase. Malgré des dizaines d’années de programmes de lutte gouvernementaux, la schistosomiase reste une important problème de santé publique dans le pays. Des milliers de personnes sont infectées chaque année par le trématode, et des millions habitent dans des régions d’endémie. L’Organisation mondiale de la Santé préconise l’utilisation d’une combinaison d’un molluscicide (niclosamide) et d’un traitement médicamenteux de masse pour empêcher la transmission de la schistosomiase, qui permettrait de réduire efficacement la morbidité liée à la maladie. Le niclosamide a déjà été utilisé dans les programmes officiels de lutte contre la schistosomiase au Brésil, mais *B. glabrata* regagne du terrain après les traitements. Ce molluscicide a progressivement été abandonné et il n’est plus du tout utilisé depuis 2002, principalement à cause de pressions mondiales croissantes pour la protection de l’environnement et de la difficulté d’obtenir des autorisations du Ministère de l’Environnement brésilien pour l’épandage de substances toxiques dans les écosystèmes aquatiques. Il est donc nécessaire de développer de nouveaux molluscicides qui viseraient plus sélectivement *Biomphalaria* et seraient moins nocifs pour le milieu aquatique. Par ailleurs, des efforts politiques sont nécessaires pour convaincre les bailleurs de fonds de financer ce domaine de recherche. Dans ce contexte, le présent article présente une analyse critique de l’utilisation de molluscicides dans les programmes de lutte contre la schistosomiase au Brésil.

Translated from English version into French by Suzanne Assenat, through

TRANSLATORS
WITHOUT BORDERS

Критический анализ моллюскоцидного использования в программах контроля шистосомиаза в Бразилии

PMZ Коэльо и RL Кальдеира

Резюме

В Бразилии улитки *Biomphalaria glabrata*, *B. tenagophila*, и *B. straminea* естественным образом заражаются трематодом *Schistosoma mansoni*, вызывающим шистосомиаз. Несмотря на десятилетия усилий, прилагаемых правительством в официальных программах контроля, шистосомиаз остается важной проблемой здоровья в стране; тысячи людей заражаются трематодом ежегодно и миллионы живут в эндемических зонах. Всемирная Организация здравоохранения рекомендует использовать сочетание моллюскоцида (никлозамида) и массовой химиотерапией для контроля передачи шистосомиаза, так как это лечение успешно сокращает процент заболеваемости. Раньше никлозамид использовался в Бразилии в официальных программах контроля шистосомиаза. Однако, так как улитка *B. glabrata* повторно заселяет даже после использования моллюскоцида, их использование в стране значительно снизилось до полного прекращения в 2002 году, в основном по причине возрастающего глобального давления касательно защиты

окружающей среды и сложностей при получении разрешений от Бразильского министерства экологии на использование токсичных веществ в водных экосистемах. Таким образом, необходима разработка новых моллюскоцидов, которые могли бы быть более селективными по отношению к видам улиток *Биомфалярий* и менее вредным для водных экосистем. К тому же требуется усилия со стороны политиков для привлечения внимания инвесторов для предоставления грантов этой области исследований. Для этих целей настоящая статья нацелена на предоставление критического анализа использования моллюскоцидов в программах контроля шистосомиаза в Бразилии.

Translated from English version into Russian by Anna Haas, through

Análisis crítico de la aplicación de molusquicidas en programas de control de la esquistosomiasis en Brasil

PMZ Coelho and RL Caldeira

Resumen

En Brasil, *Biomphalaria glabrata*, *B. tenagophila* y *B. straminea* se ven infectados de forma natural por el trematodo *Schistosoma mansoni*, que es el agente causante de la esquistosomiasis. Pese a décadas de esfuerzos por parte del gobierno a través de programas oficiales de control, la esquistosomiasis sigue siendo un importante problema de salud pública en el país: miles de personas se infectan con el trematodo cada año y millones de personas viven en zonas endémicas. La Organización Mundial de la Salud recomienda utilizar una combinación de molusquicida (niclosamida) y quimioterapia intensiva para controlar la transmisión de la esquistosomiasis ya que este tratamiento reduce con éxito la morbilidad de la enfermedad. En el pasado se ha utilizado la niclosamida en programas oficiales de control de esquistosomiasis en Brasil. Sin embargo, dado que *B. glabrata* se recoloniza incluso tras la aplicación del molusquicida, el uso de molusquicidas ha disminuido gradualmente en el país hasta que se dejó de utilizar definitivamente en 2002, debido principalmente a la creciente presión a escala mundial por la conservación del medio ambiente y las dificultades para obtener los permisos del Ministerio de Medio Ambiente brasileño para el uso de sustancias tóxicas en ecosistemas acuáticos. Por tanto es necesario descubrir nuevos molusquicidas que puedan ser más selectivos para las especies de *Biomphalaria* y menos perjudiciales para el ecosistema acuático. Además se necesita dedicar esfuerzos a la política de sensibilización de los donantes que proporcionen fondos para este campo de investigación. En este contexto, este artículo trata de ofrecer un análisis crítico de la aplicación de molusquicidas en los programas de control de esquistosomiasis en Brasil.

Translated from English version into Spanish by SergioLorenzi, through
